# Getting to Implementation: applying data-driven implementation strategies to improve guideline concordant surveillance for hepatocellular carcinoma

**DOI:** 10.1186/s13012-025-01469-w

**Published:** 2025-12-12

**Authors:** Vera Yakovchenko, Chaeryon Kang, Brittney Neely, Carolyn Lamorte, Heather McCurdy, Dawn Scott, Anna Nobbe, Gwen Robins, Nsikak R. Ekanem, Monica Merante, Sandra Gibson, Patrick Spoutz, Linda Chia, Rachel I. Gonzalez, Matthew J. Chinman, David Ross, Maggie Chartier, Lauren A. Beste, Jasmohan S. Bajaj, Tamar Taddei, Timothy R. Morgan, Shari S. Rogal

**Affiliations:** 1 Center for Healthcare Evaluation, Research, and Promotion, VA Pittsburgh Healthcare System, Building 30, Room 2A113 University Drive C (151C, Pittsburgh, PA 15240 USA; 2 Department of Psychiatry, University of Pittsburgh, Pittsburgh, PA USA; 3 Gastroenterology Section, VA Ann Arbor Healthcare System, Ann Arbor, MI USA; 4 Department of Medicine, Central Texas Veterans Healthcare System, Temple, TX USA; 5 Digestive Diseases Section, Cincinnati VA Medical Center, Cincinnati, OH USA; 6 Gastroenterology Section, Martinsburg VA Medical Center, Martinsburg, WV USA; 7 VA Northern Indiana Healthcare System, Fort Wayne, IN USA; 8 Division of Gastroenterology, Hepatology, and Nutrition, University of Pittsburgh, Pittsburgh, PA USA; 9 Pharmacy Benefits Management, Veterans Integrated Service Network 20, Vancouver, WA USA; 10 Research Service, VA Long Beach Healthcare System, Long Beach, CA USA; 11 RAND Corporation, Pittsburgh, PA USA; 12 Specialty Care Program Office, Veterans Health Administration, Washington, DC USA; 13 General Medicine Service, VA , Puget Sound Health Care System, Seattle, WA USA; 14 Division of General Internal Medicine, Department of Medicine, University of Washington School of Medicine, Seattle, WA USA; 15 Gastroenterology Section, Richmond VA Health Care System, Richmond, VA USA; 16 Division of Gastroenterology, Hepatology and Nutrition, Virginia Commonwealth University, Richmond, VA USA; 17 VA Connecticut Healthcare System, West Haven, CT USA; 18 Section of Digestive Diseases, Department of Internal Medicine, Yale School of Medicine, New Haven, CT USA; 19 National Gastroenterology and Hepatology Program, Veterans Health Administration, Washington, DC USA; 20 Gastroenterology Section, VA Long Beach Healthcare System, Long Beach, CA USA; 21 Division of Gastroenterology, Department of Medicine, University of California, Irvine, CA USA; 22 Department of Surgery, University of Pittsburgh, Pittsburgh, PA USA; 23 Michael DeBakey VA Medical Center, Galveston, TX USA

**Keywords:** Cirrhosis, Intervention, Dissemination, Cancer, Hepatoma

## Abstract

**Background:**

While guidelines recommend twice-yearly liver cancer (hepatocellular carcinoma, HCC) surveillance for people with cirrhosis, adherence to these guidelines remains variable. We aimed to empirically identify and apply successful implementation strategies through Getting to Implementation (GTI), a manualized facilitation approach.

**Methods:**

A hybrid type III, stepped-wedge, cluster-randomized trial was conducted at 12 underperforming Veterans Health Administration (VA) sites between October 2020 and October 2022. GTI included a stepwise approach to guide sites to detail their current state, set implementation goals, identify implementation barriers, select implementation strategies, make a work plan, conduct an evaluation, and sustain their work. Outcomes were defined using the *Reach*, *Effectiveness*, *Adoption*, *Implementation*, and *Maintenance* (RE-AIM) framework.

**Results:**

Facilitators supported site teams with an average of 20±6 facilitation hours over a 12-month period. Ten of 12 sites (83%) adopted GTI and applied a median of five strategies (e.g., dashboard use, small tests of change, direct patient outreach). *Reach*, the primary outcome, increased from mean 29.1% to mean 38.8% at-risk Veterans receiving HCC surveillance from pre- to post-intervention, and further increasing to 41.3% in the sustainment period. In both unadjusted and adjusted models, the odds of HCC surveillance were significantly higher during intervention (adjusted odds ratio, aOR=1.67, 95% CI:1.59, 1.75) and during sustainment (aOR=1.69, 95% CI:1.60, 1.78) compared with baseline, and with difference between active and sustainment periods, indicating sustained improvement after active facilitation ended.

**Conclusions:**

GTI sustainably improved HCC surveillance, suggesting that applying data-driven implementation strategies within a manualized facilitation approach can improve care.

**Clinical Trial Registration:**

ClinicalTrials.gov**,** NCT04178096

**Supplementary Information:**

The online version contains supplementary material available at 10.1186/s13012-025-01469-w.

Contributions to the literature

- Applying data-driven strategies is an important research priority to optimize the scalability of implementation science efforts.
- This study explored whether underperforming healthcare sites could improve liver cancer surveillance rates through applying data-driven strategies via a manualized facilitation approach (Getting to Implementation, GTI).
- Results showed sustainably improved liver cancer surveillance, with sites implementing a median of five data-driven strategies and a strong association between fidelity to GTI and relative change in screening performance.
- Next steps involve elucidating mechanisms of change across implementation phases and continuing to adapt our approach to new contexts.

## Introduction

Surveillance for liver cancer (hepatocellular carcinoma, HCC) is widely recommended by US and international guidelines but has been variably applied across healthcare systems [1–5]. HCC surveillance rates in the Veterans Health Administration (VA) range widely from 30% to 70% across its 140 medical centers (surpassing non-VA rates of as low as 7%) [2]. Such variation suggests both local (e.g., staffing, culture) and system-level (e.g., policy, metrics) implementation barriers, extending beyond patient-related factors [5–11]. Thus, healthcare systems often need both system-facing and locally tailored strategies to address barriers and improve surveillance rates.

Implementation strategies are methods to enhance the adoption, implementation, or sustainment of evidence-based practices and programs (like HCC surveillance) [12]. These strategies range broadly, from policy changes to clinician education support, digital tools, or complex strategies that include the ongoing guidance of an implementation expert. The Expert Recommendations for Implementing Change (ERIC) project offers a taxonomy of strategy names and definitions, allowing researchers to measure strategy use across projects and sites [13].

Implementation science researchers aim to develop approaches to simultaneously identify precise strategies that can address implementation barriers and lead to success for underperforming sites and apply them in replicable and scalable ways. However, it has been challenging to develop a widely applicable approach for selecting implementation strategies from among 73 choices in the ERIC taxonomy. Many described approaches require expertise in the areas of behavior change theory and implementation science [14, 15]. Such theory-driven and mechanistically based approaches are often highly effective but are also challenging to scale. Data-driven approaches have aimed to use surveys to identify combinations of strategies associated with improved implementation outcomes [16–20]. While these “high-value” or “data-driven” strategies are promising, it has been unclear whether strategies that are supported by observational data can be applied to other settings where implementation is poor.

To address this need in implementation science, we developed Getting to Implementation (GTI) as an implementation playbook that packaged data-driven implementation strategies for improving HCC surveillance. GTI is an adaptation of Getting To Outcomes (GTO), a 10-step process for selecting behavioral interventions to apply, often in community settings (i.e., teen pregnancy prevention efforts) [21–24]. GTI is a stepwise approach to select, apply, and evaluate implementation strategies in a clinical setting. We previously summarized the strategies that were found to be associated with HCC surveillance across VA [25, 26], yet it was unclear whether such strategies could be applied to improve care in lower performing VAs. Therefore, this trial aimed to assess the effectiveness of applying data-driven implementation strategies through a facilitated intervention (GTI) to increase HCC surveillance.

## Methods

### Design

This hybrid type III implementation-effectiveness, stepped-wedge, cluster randomized trial was conducted in the VA. Hybrid-type III trials focus primarily on implementation and the extent to which implementation strategies work to impact care delivery, with a secondary focus on effectiveness for improving clinical outcomes. Per regulations outlined in VHA Program Guide 1200.21, this project was deemed a non-research operations activity. All participation was voluntary. Standards for QUality Improvement Reporting Excellence (SQUIRE) 2.0 guidelines were used for reporting of this study [27]. All authors had full access to the study data and reviewed and approved the final manuscript.

### Setting, Participants, and Randomization

The VA healthcare system is composed of over 140 VA Medical Centers (hereafter sites) across the US. Prior to beginning the trial, power calculations indicated that, using stepped-wedge design, 12 sites receiving GTI would be adequately powered to detect a clinically meaningful effect size of 0.05 (Cohen’s d) [28]. For this trial, we recruited 16 sites from the bottom quartile of performance on baseline assessments of HCC surveillance rates. Veterans were assigned to the site of their primary care provider, or, in cases where they did not receive primary care, where they had the most documented encounters. As per stepped-wedge methods, sites were randomized as to the timing of starting the intervention (i.e., round), with four sites crossing from control to intervention every six months, until all sites were exposed from October 2020–October 2021 (with implementation being completed in October 2022 and observation continued through October 2023). Block set randomization was done by random number generation at the site level and accounted for volume (using a threshold of 400 Veterans with cirrhosis), site complexity [29], and availability of on-site gastroenterology (GI) specialty care (vs. not).

### Implementation strategies

GTI is a multi-step intervention that includes how to build a team, identify the problem and goals, prioritize barriers to implementation, select implementation strategies, make a work plan to implement strategies, evaluate progress, and iterate and sustain this work. These GTI steps are embedded with first- and second-level implementation strategies. First-level strategies are inherent to GTI and include building an interdisciplinary team, assessing for readiness and identifying barriers and facilitators, developing an implementation blueprint, conducting audit and feedback, and purposefully reexamining implementation.

Second-level implementation strategies were empirically derived based on annual surveys of implementation strategy use across all VA sites and their relationship to guideline-concordant cirrhosis care. This process has been previously described in detail [25]. The following second-level strategies were available for selection by local site team members: 1) learning collaborative, 2) population health management dashboard, 3) direct patient outreach, 4) clinical expert consultation, 5) clinician education, 6) electronic clinical reminder alert in the healthcare record, 7) tailoring care, and 8) small tests of change [25]. These strategies were detailed in the GTI playbook in accordance with recommendations of Proctor et al., including the mechanism/purpose, actor, dose of the strategy, as well as the core vs. tailorable components [12]. Facilitators also recorded sites’ use of other strategies not pre-specified by GTI. These were categorized using the ERIC taxonomy and added to the second-level strategy set used by sites.

GTI is facilitated by a clinical facilitator and evaluation facilitator dyad, where facilitators guide a site implementation team through the GTI steps. Clinical facilitators included four Advanced Practice Providers (APPs), one Clinical Pharmacy Specialist, and one registered nurse with expertise in hepatology and leadership roles in a national hepatology learning collaborative. Evaluation facilitators included two social workers with quality improvement and research training. At each participating site, local site leaders referred clinicians and staff with relevant experience as potential members of the site implementation team. The core site implementation team was a subset of individuals who had specialized knowledge of HCC surveillance processes at their site and were continually involved in GTI. The clinical/evaluation facilitator dyad met with site implementation team members for six months every two weeks for one hour on a virtual platform to work through the GTI playbook and worksheets [28]. Following this six-month active implementation period was six months of sustainment with ad hoc (but at least monthly) facilitation meetings, as requested by site teams.

### Data Collection and Measures

#### Covariates

Patient-level data were collected from the Corporate Data Warehouse (CDW) and included Veteran demographic characteristics (age, race/ethnicity, gender, rurality), socioeconomic (Area Deprivation Index), and health characteristics. Rurality (urban versus rural/highly rural) was based on standard Rural-Urban Commuting Area classification using home address [30]. Area Deprivation Index is a validated measure of neighborhood disadvantage and was determined using the Neighborhood Atlas resource, where people are given a score based on their zip codes [31]. Comorbidities were assessed using two outpatient or one inpatient diagnosis code(s) or listing(s) in the problem list. Etiology and severity of liver disease were ascertained using diagnosis codes for common etiologies (e.g., alcohol, viral hepatitis), as well as codes for hepatic decompensation events (e.g., ascites, encephalopathy, hepatorenal syndrome). The Model for End Stage Liver Disease-Sodium (MELD-Na) is a score developed to categorize patients with cirrhosis. Increased MELD-Na is associated with mortality and was calculated using laboratory values for sodium, creatinine, bilirubin, and international normalized ratio, collected as close to the baseline as possible [32]. Platelets, an independent marker of portal hypertension, were collected from the laboratory data in CDW. Site-level variables included site complexity (a VA composite score incorporating several site-level factors including patient load and acuity, research funding, the availability of specialty care, and location; Level 1 high complexity vs. Level 2 medium and level 3 low complexity;) [29] and geographic Census region [33].

#### Implementation

Each facilitation event was tracked by date, medium (message, email, phone), time spent (dose), team members involved and who initiated the contact (site or facilitator). Barriers, facilitators, adaptations, and needed follow-up were also tracked for each event. Events were coded by the types of primary and secondary activities conducted using a standardized time tracking method that included 16 facilitation activities assigned to the three implementation phases (pre-implementation, implementation, sustainment) [34]. For example, “Action/Implementation Planning” was categorized as a pre-implementation activity, “Problem-Solving” as implementation, and “Pulling Back and Letting Sites Lead” as sustainment. 

Evaluation facilitators tracked second-level strategy selection, use, and timing by site teams at baseline, 6 months, and 12 months using meeting notes and direct observation. Fidelity to the GTI process (e.g., low, medium, high) was defined as the degree to which site implementation teams completed GTI tools, attended scheduled meetings, and stayed within the intervention timeline of six months. 

#### Outcomes

The RE-AIM evaluation framework states that an evidence-based practice only improves public health to the extent which it can *Reach* the target population, and is *Effective*, *Adopted* by users, *Implemented* with fidelity, and *Maintained *(hereafter Sustainment) [35]. The primary implementation outcome was site-level adoption of GTI, defined as the site initiating and completing all steps of GTI. Successful adoption at the study level was defined a priori as 80% of sites that started GTI reaching this endpoint [36]. Fidelity to the GTI process as described above was categorized as low, medium or high.

Given that HCC surveillance is already known to be effective and has tightly prescribed implementation, the primary clinical outcome was reach of HCC surveillance, defined as the patient-level receipt of an ultrasound or contrasted CT or MRI within the prior six months among Veterans with cirrhosis. A secondary clinical outcome was sustainment, defined as surveillance rates at 24 months (or 12 months post intervention). The denominator included only Veterans who were engaged in care at the site of interest, defined as having at least one encounter for clinical care in the prior 18 months. These data were collected from the VA Corporate Data Warehouse every 6 months, following stepped-wedge methods.

### Analysis

Data were analyzed using intent-to-treat principles, such that Veterans were assigned to a site at the start of the study and then included in analysis (other than one participating site without data available due to transition to the Cerner electronic health record mid-trial). The primary question was whether GTI was associated with improved HCC surveillance rates at the end of intervention (12 months) and at 24 months. This was assessed using generalized linear mixed models with logistic regression. We first modeled the association between GTI and HCC surveillance without adjustment for individual- or site/cluster-level covariates. Subsequently, we adjusted for individual- and cluster-level covariates, and then did sensitivity analyses to assess whether removing early pandemic period (January 1, 2020 – September 30, 2020) data impacted models. These models all assessed the patient-level binary outcome of receipt of HCC surveillance in the 6-month period. Initially we specified three random effects: the random effect for the repeated measures from individual *i* in cluster *k*, the random cluster-by-time interaction, and the within-cluster intraclass correlation coefficient (ICC). The estimated variance of the random effect related to the cluster-by-time interaction was nearly zero, indicating that a random effect was unnecessary, and a constant effect sufficed. Note that the assignment of interventions is confounded with time in the stepped-wedge, cluster-randomized trials, so modeling the background secular trend is important to remove the bias in estimating the effect attributed solely to the intervention. We assumed that the average secular trend is a distinct value during each period, and we used a nonparametric representation for the time effect (categorical time).

## Results

### Site and Participant characteristics

Of 16 sites invited to participate in GTI, 12 sites agreed to participate (75%; three did not respond to the invitation and one site lead did not believe in the evidence enough to warrant improvement efforts), and 10 sites completed all steps of GTI (83%). The 12 enrolled sites represent various levels of facility complexity (four high complexity, five medium complexity, and three low complexity), and range in patient volume and various patient characteristics. Table 1 shows the characteristics of 8,961 Veteran patients with cirrhosis receiving care in the intervention sites, by round, compared to the characteristics of the patients with cirrhosis across VA nationally (*n*=108,896). Both patient- and site-level differences between GTI rounds were observed and later analyzed in multi-variable analysis.

**Table 1:** Patient and site-level characteristics (n=108,896)

	**Total**	**No GTI**	**GTI Round 1**	**GTI Round 2**	**GTI Round 3**	***p*-value**
	*N* = 108,896	*N* = 99,935	*N* = 3,658	*N* = 3,328	*N* = 1,975	
Demographic Characteristics						
Age (SD)	66.35 (9.69)	66.32 (9.67)	66.85 (9.56)	66.62 (10.25)	66.44 (10.01)	0.003
Male Sex	103,922 (95%)	95,404 (95%)	3,452 (94%)	3,174 (95%)	1,892 (96%)	
Race and Ethnicity						<0.001
Hispanic	8,776 (8.1%)	7,765 (7.8%)	382 (10%)	102 (3.1%)	527 (27%)	
Non-Hispanic Black	19,494 (18%)	18,541 (19%)	337 (9.2%)	495 (15%)	121 (6.1%)	
Non-Hispanic White	72,635 (67%)	66,307 (66%)	2,647 (72%)	2,513 (76%)	1,168 (59%)	
Other/Unknown	5,597 (5.1%)	5,075 (5.1%)	222 (6.1%)	161 (4.8%)	139 (7.0%)	
Rural	36,306 (33%)	33,983 (34%)	711 (19%)	1,061 (32%)	551 (28%)	<0.001
Area Deprivation Index	58.11 (25.51)	57.83 (25.72)	56.87 (21.13)	62.13 (23.71)	68.15 (22.22)	<0.001
Homeless or unstable housing	25,881 (24%)	23,905 (24%)	799 (22%)	762 (23%)	415 (21%)	<0.001
Liver-related Characteristics						
Etiology of Liver Disease						<0.001
Hepatitis C	34,154 (31%)	31,698 (32%)	1,113 (30%)	806 (24%)	537 (27%)	
Alcohol-related Liver Disease	40,902 (38%)	37,326 (37%)	1,418 (39%)	1,439 (43%)	719 (36%)	
MASH	13,966 (13%)	13,095 (13%)	455 (12%)	228 (6.9%)	188 (9.5%)	
Other*	2,281 (2.1%)	2,100 (2.1%)	70 (1.9%)	73 (2.2%)	38 (1.9%)	
None	17,593 (16%)	15,716 (16%)	602 (16%)	782 (23%)	493 (25%)	
Platelet Count (SD)	174.10 (80.26)	174.02 (80.01)	176.13 (80.79)	175.68 (87.22)	171.46 (79.42)	0.200
MELD-Na (SD)	10.67 (4.90)	10.66 (4.91)	10.87 (4.85)	10.96 (4.83)	10.34 (4.55)	<0.001
Decompensated Cirrhosis	32,281 (30%)	29,870 (30%)	976 (27%)	997 (30%)	438 (22%)	<0.001
GI visit in the last year	57,292 (53%)	54,215 (54%)	1,586 (43%)	967 (29%)	524 (27%)	<0.001
Comorbidities						
Charlson Score (SD)	2.90 (1.85)	2.91 (1.86)	2.68 (1.71)	2.86 (1.89)	2.63 (1.68)	<0.001
Alcohol Use Disorder	49,657 (46%)	45,667 (46%)	1,589 (43%)	1,599 (48%)	802 (41%)	<0.001
Substance Use Disorder	17,286 (16%)	16,168 (16%)	457 (12%)	441 (13%)	220 (11%)	<0.001
Mental Health Disorder	67,750 (62%)	62,238 (62%)	2,287 (63%)	1,997 (60%)	1,228 (62%)	0.065
Facility Characteristics						
Complexity						<0.001
1a-highest	51,092 (47%)	49,548 (50%)	0 (0%)	1,544 (46%)	0 (0%)	
1b	20,602 (19%)	18,351 (18%)	2,251 (62%)	0 (0%)	0 (0%)	
1c	20,836 (19%)	20,159 (20%)	0 (0%)	677 (20%)	0 (0%)	
2	8,357 (7.7%)	6,129 (6.1%)	575 (16%)	464 (14%)	1,189 (60%)	
3-lowest	7,941 (7.3%)	5,680 (5.7%)	832 (23%)	643 (19%)	786 (40%)	
Census Region						<0.001
Northeast	13,847 (13%)	12,086 (12%)	982 (27%)	0 (0%)	779 (39%)	
Midwest	20,275 (19%)	19,632 (20%)	0 (0%)	643 (19%)	0 (0%)	
South	50,675 (47%)	45,007 (45%)	2,251 (62%)	2,221 (67%)	1,196 (61%)	
West	24,099 (22%)	23,210 (23%)	425 (12%)	464 (14%)	0 (0%)	

^*^ = PSC, PBC, AIH, Hemochromatosis, Hepatitis B

Site implementation teams included seven to 32 total participants, for an overall total of 200 participants ever attending a GTI meeting. The core site implementation teams were composed of two to eight individuals and totaled 53 people across sites. Leadership members were involved at every site. All but one site had physicians involved, eight had APPs, and seven had clinical pharmacy specialists. Nine of 12 teams had GI specialists involved, seven primary care, six radiology/imaging, and fewer had pharmacy, community care, and behavioral health involved. 

### Primary outcome

**Fig. 1:**
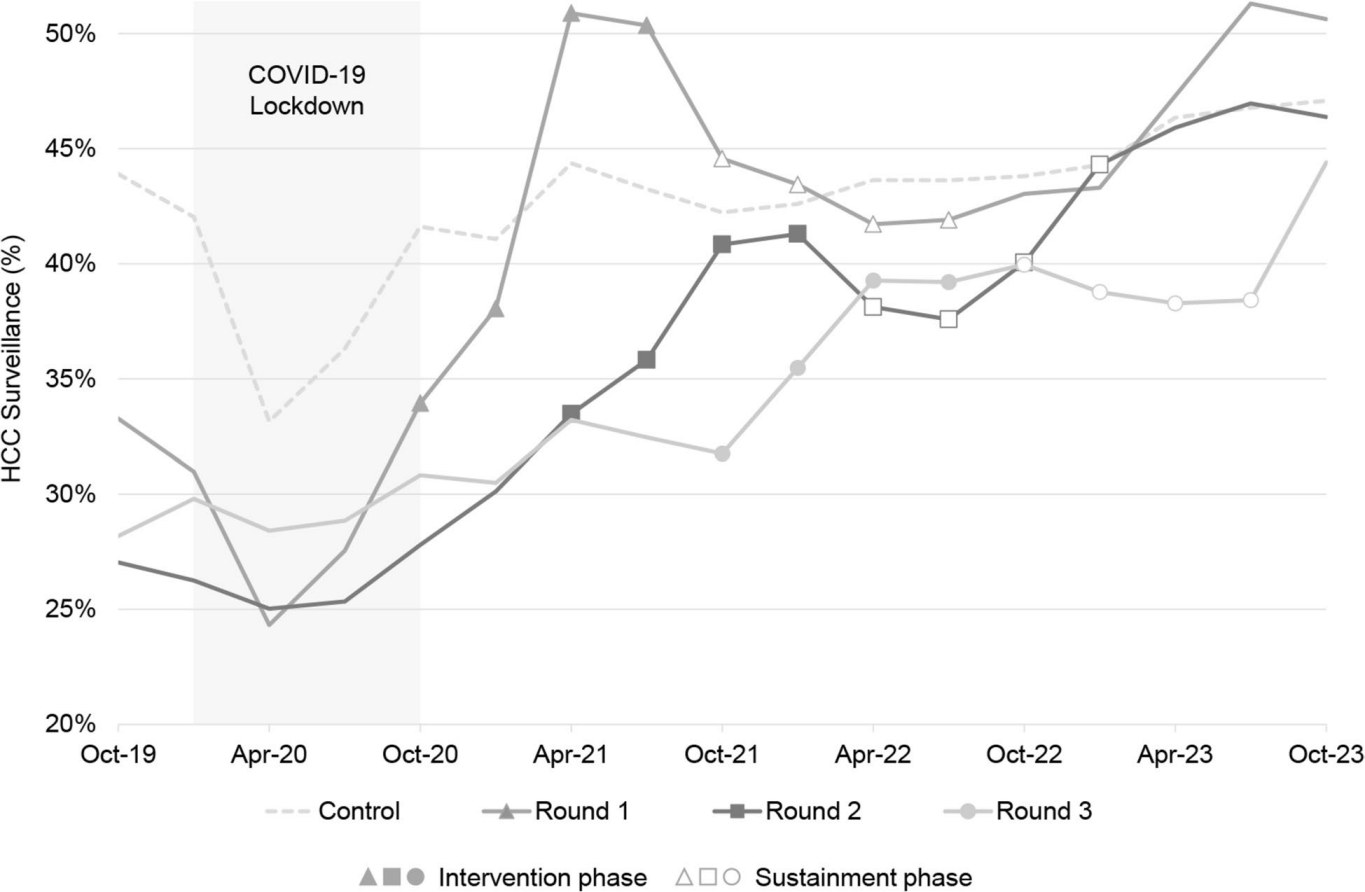
HCC surveillance over time, by stepped-wedge randomization round

**Fig. 2:**
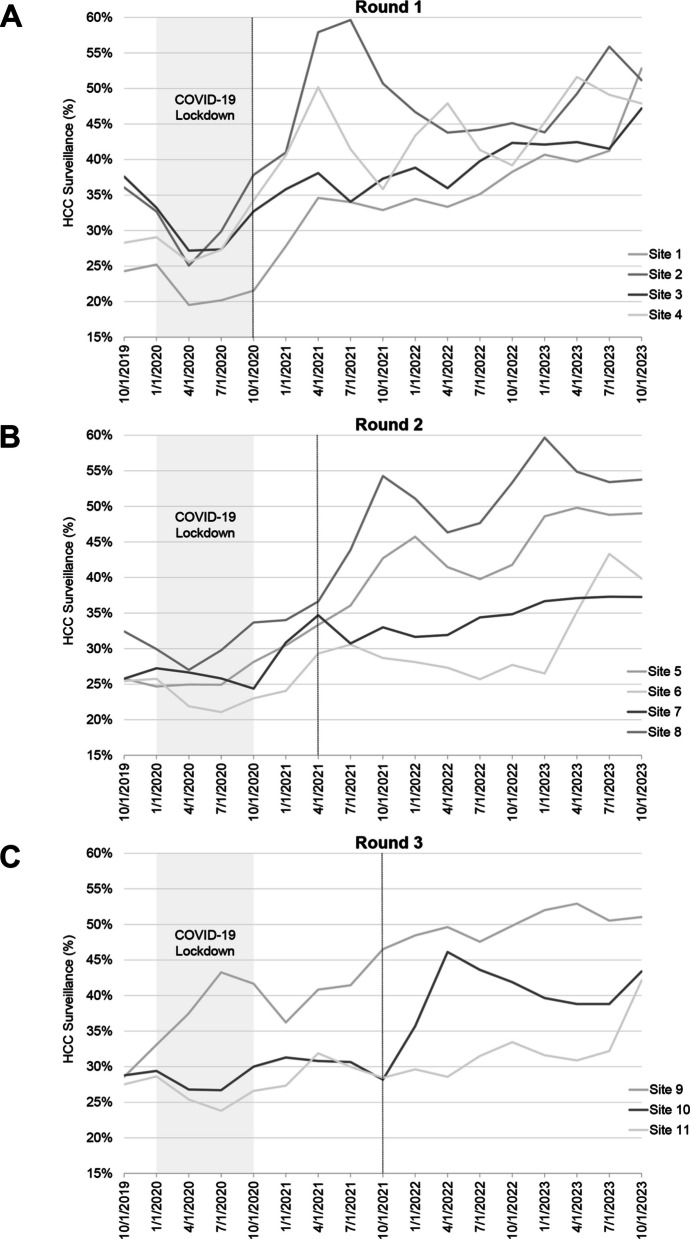
Site-level HCC surveillance rate over time, by stepped-wedge randomization arm

The mean baseline HCC surveillance rate among intervention sites was 29.1% (median 29.2%, range 23.9%–37.7%), and this increased to mean 38.8% (median 38.5%, range 29.3%–49.8%) during the intervention and continued to increase during sustainment (mean 41.3%, median 40.4%, range 29.2%–52.6%). The primary outcome of HCC surveillance is illustrated in Fig. 1. Because the sites were randomized to their GTI start time, the timing of improvement in HCC surveillance was expected to correspond to the timing of intervention. Figure 2 displays trends over times of each site by round. Sites 6 and 12 did not complete the steps of GTI, and Site 12 was excluded from the intent to treat analysis due to its transition to VA’s new electronic medical record and lack of available data.

The unadjusted model (Table 2) demonstrates that GTI was associated with a significant increase in HCC surveillance at the end of the intervention (OR=1.67, 95% CI=1.59, 1.75) and in the sustainment period (OR=1.68, 95% CI=1.60, 1.76), with no significant drop-off in effect from intervention to sustainment (*p*=0.834). After controlling for the effect of individual-level and cluster-level covariates, the odds of HCC surveillance were significantly higher with GTI vs. control (aOR=1.67, 95% CI=1.59, 1.75), and with sustainment compared to the control (aOR=1.69, 95% CI=1.61, 1.78). The odds of HCC surveillance during active implementation (12 months) were not significantly different from those in sustainment (24 months). The estimated within-person ICC and within-site ICC were 0.48 and 0.03.

**Table 2:** Unadjusted model of GTI treatment effect, during intervention and sustainment

	**Estimates**	**95% CI**	***P* value**
Treatment effect change during active implementation vs. control	1.67	[1.59, 1.75]	<0.001
Treatment effect change under the sustainment vs. control	1.68	[1.60, 1.76]	<0.001
Active implementation vs. Sustainment	1.00	[0.96, 1.05]	0.834

### Covariate effects

Factors significantly associated with HCC surveillance in adjusted models included age, race/ethnicity, rurality, Area Deprivation Index (ADI), and region of the country (Table 3). Age at baseline was negatively associated with HCC surveillance (aOR=0.99; 95% CI=0.99, 1.00). HCC surveillance was lower in non-Hispanic Black or African American Veterans with cirrhosis (aOR=0.94, 95% CI=0.91, 0.98) and Veterans of other or unknown race (aOR=0.85, 95% CI=0.80, 0.90), and higher among Veterans who were Hispanic or Latino (aOR=1.15, 95% CI=1.09, 1.22), compared to non-Hispanic white Veterans. HCC surveillance was lower among Veterans in rural areas (aOR=0.92, 95% CI=0.89, 0.95), ADI (national rank) was negatively associated with HCC surveillance (aOR=0.99; 95% CI=0.99, 1.00). Odds of HCC surveillance were significantly higher in the Midwest (aOR=1.40; 95% CI=1.09, 1.81) and West (aOR=1.46; 95% CI=1.13, 1.89) vs. the Northeast region.

**Table 3:** Association between HCC surveillance and covariates, adjusted model

**Covariate**	**OR**	**95% CI**	***P* value**
Age	0.99	[0.99, 0.99]	<0.001
Sex (vs. men)			
Women	0.95	[0.89, 1.01]	0.101
Transgender	1.10	[0.81, 1.50]	0.529
Race/Ethnicity (vs. non-Hispanic White)			
Non-Hispanic Black or African American	0.94	[0.91, 0.98]	0.002
Hispanic or Latino	1.15	[1.09, 1.22]	<0.001
Other/Unknown	0.85	[0.80, 0.90]	<0.001
Rural (vs. urban)	0.92	[0.89, 0.95]	<0.001
ADI (national rank)	0.99	[0.99, 1.00]	0.019
Census region (vs. Northeast)			
Midwest	1.46	[1.13, 1.89]	0.004
South	1.08	[0.86, 1.36]	0.494
West	1.40	[1.09, 1.81]	0.009
GTI (vs. control)			
Implementation (year 1)	1.67	[1.59, 1.75]	<0.001
Sustainment (year 2)	1.69	[1.61, 1.78]	<0.001

*Abbreviations*: *ADI* Area deprivation index, *GTI* Getting to implementation

### Impact of the COVID-19 pandemic

To explore the sensitivity of the interference due to the COVID-19 pandemic, we repeated the analysis by excluding time from January 1, 2020, to September 30, 2020. The odds of HCC surveillance during GTI (OR=1.70, 95% CI=1.60, 1.80, *p*-value <0.001) and sustainment (OR=1.70, 95% CI=1.60, 1.80, *p*-value <0.001) were significantly increased compared to the control. No significant difference was observed between the active implementation and sustainment groups (OR=1.00, 95% CI=0.96, 1.05, *p*-value = 0.95), indicating a stronger positive association between the GTI and HCC surveillance when the pandemic-related data were excluded. Likewise, the adjusted model reflected these findings. The adjusted odds of HCC surveillance were higher with active GTI vs. control (aOR=1.71; 95% CI=1.61, 1.82) and sustainment vs. control (aOR =1.74, 95% CI=1.64, 1.85). Covariate associations between HCC surveillance remained unchanged in these sensitivity models.

### Facilitation and fidelity

Over the 12 months of GTI, facilitators held an average of 25 live meetings with sites (range 16–49). In total, sites received an average of 20±6 hours of facilitation (range 11–29) across an average of 68±18 facilitation events (range 39–111), which included live meetings, emails, instant messages, and phone calls. Sites averaged 12±8 site-initiated contacts, accounting for 4–45% of all contacts (average 24%).

Facilitation dose (defined as the amount of time facilitators engaged with sites) and site-initiated facilitation support were not associated with HCC surveillance. However, the frequency of specific types of facilitation activities signaled potential differences, as sites with more implementation- and sustainment-oriented activities (33.0% and 44.2%, respectively) had greater HCC improvement compared to pre-implementation activities (16.9%, *p*=0.23). Fidelity to the GTI process was associated with an increase in HCC surveillance: low fidelity sites increased by an average of 15.4%, medium fidelity sites by 27.6%, and high fidelity sites by 47.1% (*p*=0.009).

### Strategy implementation

Table 4 shows the original eight pre-specified second-level strategies and three non-pre-specified strategies used by the 12 sites. At baseline, sites reported applying a median of two of the eight pre-specified strategies, with “consulting experts” and “reaching out to Veterans with cirrhosis” being the most commonly reported. During GTI, sites began using a median of five additional pre-specified second-level strategies. The most commonly selected new strategies were “educate clinicians” (100%), “use a population health management dashboard” (92%), “participate in a learning collaborative” (75%), and “use a clinical reminder” (67%).

**Table 4:** Strategy adoption by site

	**Round 1**				**Round 2**				**Round 3**			
**Strategies**	**Site 1**	**Site 2**	**Site 3**	**Site 4**	**Site 5**	**Site 6**	**Site 7**	**Site 8**	**Site** **9**	**Site** **10**	**Site** **11**	**Site** **12**
Work with the National Learning Collaborative	◻	◻	◻	▲	▲	▲	▲	▲	▲	▲	▲	▲
Use the cirrhosis dashboard	▲	▲	-	▲	▲	▲	▲	▲	▲	▲	▲	▲
Reach out directly to Veterans	▲	▲	◻	▲	◻	▲	▲	▲	◻	◻	◻	◻
Consultation with cirrhosis experts	◻	◻	◻	◻	◻	◻	◻	◻	◻	◻	▲	▲
Provide ongoing training to local clinicians	▲	▲	▲	▲	▲	▲	▲	▲	▲	▲	▲	▲
Use the HCC clinical reminder	◻	▲	▲	-	▲	▲	▲	◻	◻	▲	▲	▲
Tailor strategies to deliver care	-	-	▲	◻	-	◻	◻	-	▲	-	▲	-
Conduct small tests of change	▲	▲	-	▲	-	▲	▲	-	▲	▲	-	-
Add HCC to performance appraisal	▲	-	-	-	-	-	-	-	-	-	-	-
Engage leadership	-	▲	-	▲	▲	▲	▲	▲	▲	-	▲	▲
Hire new staff	▲	▲	◻	▲	-	▲	▲	▲	▲	▲	◻	▲

Symbols: - never did; ◻ doing before GTI; ▲ started during GTI

Sites tended to focus on two strategy combinations: clinician education paired with the HCC clinical reminder, and population health management dashboard paired with outreach to Veterans with cirrhosis. Two new strategies, not included in GTI but frequently used by sites, included: “engage leadership” (75%) and “hire new staff” (*n*=9, 75%). An additional strategy, “add HCC surveillance to performance appraisal,” was used by one site (8%).

Given the sample size and high selection of strategies by sites, which created limited variability, it was not possible to identify independent associations between strategies and changes in HCC surveillance. However, several notable patterns emerged, particularly considering the timing of strategy implementation and the combinations of strategies used. For example, sites using the “tailoring” strategy prior to GTI had 48% improvement in HCC surveillance, compared to a 24% improvement among those who never attempted tailoring, and a 36% improvement among those who started tailoring during GTI (*p*=0.069). While these differences are not statistically significant, they suggest potential trends in improvement related to strategy selection and timing.

## Discussion

We evaluated the implementation and effectiveness of an intervention using a hybrid type III design stepped-wedge trial, demonstrating both high adoption and sustained improvements in HCC surveillance. These findings contribute to the growing evidence that a facilitated manualized intervention coupled with highly operationalized implementation strategies can generate meaningful improvements in evidence-based practice outcomes.

This study addressed a critical problem of how to improve cirrhosis care quality measure performance. HCC surveillance is widely recognized as an evidence-based practice that benefits patients and is recommended by hepatology guidelines [37, 38]. Despite the benefits of early cancer detection, implementation barriers such as variable perceptions about the effectiveness of HCC surveillance, concerns about costs, and logistical complexity of ensuring twice a year imaging remain, contributing to low surveillance rates [3–5, 39]. Although VA surveillance rates are substantially higher than in other settings, we identified persistent demographic differences in surveillance rates. For example, while key covariates such as race and geography were associated with differences in improvement, patients of Hispanic or Latino ethnicity had increased HCC surveillance compared to non-Hispanic or Latino patients. Future studies should examine whether tailored implementation strategies can improve access to high quality health care for all Veteran sub-populations.

Even with pandemic-related disruptions, GTI adoption and fidelity were largely successful. While the timing and patterns of HCC surveillance improvements varied across sites, most had rapid and sustained improvement into the second year post-GTI. This finding is noteworthy given the notorious challenges with sustaining research and quality impacts, particularly when external support is withdrawn [40]. Prior research suggests that sustainment is experienced by the minority of sites, with just 37% of sites sustaining a practice for two years [41].

This project found that sites with more sustainment-oriented facilitation activities achieved greater surveillance gains. Prior work has shown that Getting To Outcomes, the model adapted to create GTI, was likewise associated with sustainment [42]. Each step of GTI emphasizes sustainability, encouraging teams to consider the long term in their decisions. Measuring and ensuring sustainment is increasingly recognized as an ethical imperative for clinical and implementation research [43]. Future work should examine which specific strategies and GTI or facilitation components were related to adoption and sustainment.

While further study is needed to fully elucidate the mechanisms of change related to GTI, we hypothesize that empowering and educating implementers using a participatory approach creates sustainable change [44]. Facilitation mechanisms previously hypothesized include obtaining stakeholder acceptance, building coherence, increasing organizational capacity, changing social roles and norms, and routinizing processes [45]. Although this study was not designed to evaluate all potential mechanisms of change, we have previously found that a strong working alliance between facilitators and site team members significantly improved HCC surveillance [46]. The manualized facilitation of GTI paired with highly specific strategies likely operates on multiple levels to address behavior and system changes. First-level strategies embedded in facilitated GTI primarily focused on changing team motivation, opportunity, and capability, and second-level strategies targeted various functions and needs. This study reinforces the importance of combining implementation strategies with facilitation support, as neither alone was likely sufficient for change.

Despite GTI’s overall success, we observed site-level variation in adoption, fidelity and outcomes, consistent with other work that finds sites progress through phases of implementation at different rates, highlighting the nonlinear nature of implementation [47]. Fidelity to GTI emerged as a key to success, compared to facilitation dose which was not significantly correlated with HCC surveillance. This underscores that implementation support alone is not sufficient; rather, ensuring that the interventions are delivered as intended is essential for achieving desired outcomes.

We found no significant differences in improvement based on which implementation strategies were selected within the broader GTI intervention. This may be attributable to the relatively small sample size and limited variation in strategy selection. For example, educating clinicians was a universally selected strategy and the backbone for sites to expand on with the dashboard and direct patient outreach. We formed the recommended strategies using data from high-performing sites prior to including them in GTI, suggesting that the exact combination of these highly effective strategies is less important than their structured application. Our findings suggest that implementation strategies defined using data from ERIC surveys, when delivered in the context of this facilitated approach, can achieve meaningful and sustainable implementation.

Future studies with larger samples may be able to better delineate which strategies are most effective under different conditions. Further work is also needed to understand if strategies effective during the implementation phase are also effective for sustainment, or whether successful strategies during each phase are distinct [48].

Despite the novelty of this study and the effectiveness of GTI in improving HCC surveillance over the short and long term in the lowest performing VA sites, there are several limitations. First, the study’s completion within the VA’s nationalized healthcare system raises questions about its applicability outside of the VA. GTO has been widely applied and found cost-effective in community settings, and facilitation has likewise been successfully used outside VA [49]. Moreover, many of the data-driven strategies are potentially setting-agnostic. Since data collection from sites ended at 12 months, we were unable to monitor strategy use at the site level beyond this point. We are currently testing an adaptation of GTI for diabetes education promotion in non-VA settings and anticipate that such a model can be further exported to other settings. Another limitation stems not from the research design, but from the design of the GTI intervention. GTI may be even more usable than GTO, since 20 hours of support provided per site with GTI is nearly a quarter of the 76 hours of technical assistance required for GTO [24]. It is possible that an average of 20 hours of external support may not be scalable outside of a research context. Similarly, the first study of GTO employed a large amount of support and over the course of several studies, systematically shrunk the number of hours of support to find the least amount that would still yield an impact. The same research trajectory is recommended for GTI as we anticipate a possible scale down of contact time would not impact outcomes. To improve scalability and cost-efficiency, we have considered several models, including group GTI and a do-it-yourself (DIY) GTI. We were unable to control the impacts of the pandemic or changes in the ways in which care was delivered during the pandemic. However, we were able to *post-hoc* assess and adjust for the impacts of time using secondary analyses. Other potential limitations include the use of medical record data, which may result in the imperfect capture of our primary outcome. Another limitation stems not from the research design, but from the design of the GTI intervention. Despite these limitations, this study contributes to the growing literature demonstrating the importance of applying implementation research methods to improve hepatology care and outcomes and offers a novel approach for applying data-driven strategies to enhance care.

## Conclusions

We assessed the effectiveness of using data-driven strategies in the GTI scaffolding to improve cirrhosis management. By applying a manualized and data-driven approach to selecting implementation strategies, we achieved sustained improvements in HCC surveillance at lower performing VA sites. Given the durability of these improvements, our findings provide a strong foundation for future scaling efforts.

## Supplementary Information

Supplementary Material 1

## Data Availability

This quality improvement project was conducted as a non-research operations activity by the VA HIV, Hepatitis, and Related Conditions Programs (HHRC), with the stipulation that data would be presented in aggregate, given the sensitive information included in the dataset. However, interested parties can contact the Center for Health Equity Research and Promotion in the VA Pittsburgh Healthcare System (contact via Andrea.Krushinski@va.gov or shari.rogal@va.gov) for further inquiries or data requests.
